# Supplementary material for: Stress distribution and surface shock wave of drop impact
Source: Nat Commun. 2022 Mar 31;13:1703. doi: 10.1038/s41467-022-29345-x (PMC8971405; doi:10.1038/s41467-022-29345-x)
Supplement: Supplementary file 1 — Supplementary Information [file 41467_2022_29345_MOESM1_ESM.pdf]

Supplementary Information

Stress distribution and surface shock wave of drop impact

Ting-Pi Sun[1], Franco Álvarez-Novoa[2], Klebbert Andrade[2], Pablo Gutiérrez[3], Leonardo Gordillo[2], and Xiang Cheng[1,*]

[1]Department of Chemical Engineering and Materials Science, University of Minnesota, Minneapolis, MN 55455, USA
[2]Departamento de Física, Facultad de Ciencia, Universidad de Santiago de Chile (USACH), Santiago, Chile
[3]Instituto de Ciencias de la Ingeniería, Universidad de O'Higgins, Rancagua, Chile
[*]Email: xcheng@umn.edu

March 1, 2022

1 Continuous displacement field

We implemented the moving least squares (MLS) interpolation method to obtain a continuously differentiable displacement field from a discrete DIC displacement field of experiments [1]:

$$u(\mathbf{x}) = P^T(\mathbf{x})a(\mathbf{x}), \tag{1}$$

where $u(\mathbf{x}) = [u_r, u_z]$ is the target continuous displacement field, $P^T(\mathbf{x})$ is a polynomial basis and $a(\mathbf{x})$ is the corresponding coefficients of the basis. $\mathbf{x}$ is the continuous position vector. Because of the cylindrical symmetry of the drop-impact geometry, $\mathbf{x} = (r, z)$ with $r = 0$ and $z = 0$ at the initial impact point. The negative z direction points along the direction of the impact velocity of drops. We adopted a cubic basis $P^T = [1, r, z, r^2, z^2, rz, r^3, z^3, r^2z, rz^2]$. The

coefficients of the target function at $\mathbf{x}$ was obtained by minimizing the weighted least-square error,

$$L(\mathbf{x}) = \sum_{i=1}^{n} f(\mathbf{x}-\mathbf{b_i}) \left[P^T(\mathbf{b_i})a(\mathbf{b_i}) - w(\mathbf{b_i})\right]^2, \tag{2}$$

where w is the discrete DIC displacement field from experiments. $\mathbf{b_i}$ indicates the discrete DIC coordinates with a total $n = 540$ points in our experiments. $f(\mathbf{x}-\mathbf{b_i})$ is the weighted function defined as [2]:

$$f(\mathbf{x}-\mathbf{b_i}) = \begin{cases} \frac{\exp(1-d^2/d_m^2)-1}{e-1} & \text{for} \quad d \leq d_m \\ 0 & \text{for} \quad d > d_m \end{cases} \tag{3}$$

where $d = |\mathbf{x}-\mathbf{b_i}|$ is the distance between the continuous position $\mathbf{x}$ and $\mathbf{b_i}$. d_m is a cut-off distance $d_m = 2\sum_{i=1}^{m} |\mathbf{x}-\mathbf{b_i}|/m$. m determines the fitting range, which was fixed at $m = 43$ in this study. The minimization of Eq. (2) gives

$$u(\mathbf{x}) = P^T(\mathbf{x})a(\mathbf{x}) = P^T(\mathbf{x})A^{-1}(\mathbf{x})B(\mathbf{x})w, \tag{4}$$

where

$$\begin{aligned} A(\mathbf{x}) &= \sum_{i=1}^{n} f(\mathbf{x}-\mathbf{b_i})P(\mathbf{b_i})P^T(\mathbf{b_i}), \\ B(\mathbf{x}) &= [f(\mathbf{x}-\mathbf{b_1})P(\mathbf{b_1}), ..., f(\mathbf{x}-\mathbf{b_n})P(\mathbf{b_n})]. \end{aligned}$$

2 Impact pressure

PDMS gels are nearly incompressible with Poisson's ratio close to 0.5, which leads to a large λ. On the other hand, the bulk strain ε_b is close to 0. Therefore, the impact pressure cannot be accurately determined from the product of $\lambda\varepsilon_b$ in the linear constitutive equation

$$\sigma_{ij} = \lambda\varepsilon_b\delta_{ij} + 2G\varepsilon_{ij}, \tag{5}$$

To overcome the difficulty, we adopted the quasi-steady-state assumption [1]. Specifically, $\lambda\varepsilon_b$ is replaced by an undetermined term $-\mu$ in Eq. (5),

$$\sigma_{ij} = -\mu\delta_{ij} + 2G\varepsilon_{ij}, \tag{6}$$

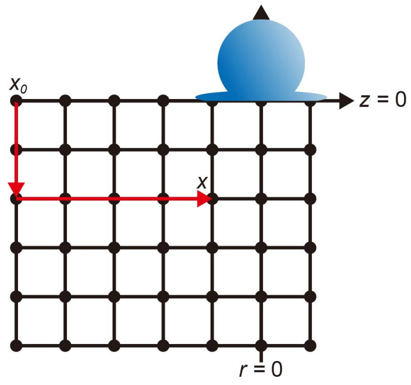

Supplementary Figure 1: Impact pressure measurements. Red arrows indicate the integration path for calculating the pressure at point $\mathbf{x}$ from the reference point at $\mathbf{x_0}$.

Under the quasi-steady-state assumption, the stress tensor obeys:

$$\sum_{j=1}^{3} \frac{\partial \sigma_{ij}}{\partial x_j} = 0. \tag{7}$$

As a result, μ can be calculated as:

$$\mu(\mathbf{x}) = \mu(\mathbf{x_0}) + G \int_{\mathbf{x_0}}^{\mathbf{x}} \left(\nabla^2 u\right) \cdot d\mathbf{s} + G \left[\varepsilon_b(\mathbf{x}) - \varepsilon_b(\mathbf{x_0})\right], \tag{8}$$

where $\mathbf{x_0}$ sets a reference point for integration. We chose the reference point at the top layer $z = 0$ with r as large as possible away from the impact point at short times. Taking $p(\mathbf{x_0}) = \sigma_{zz}(\mathbf{x_0}) = 0$, we have $\mu(\mathbf{x_0}) = 2G\varepsilon_{zz}(\mathbf{x_0})$. Finally, the pressure can be obtained

$$p(\mathbf{x}) = -G \int_{\mathbf{x_0}}^{\mathbf{x}} \left(\nabla^2 u\right) \cdot d\mathbf{s} + G \left[\varepsilon_b(\mathbf{x}) - \varepsilon_b(\mathbf{x_0})\right] + 2G \left[\varepsilon_{zz}(\mathbf{x}) - \varepsilon_{zz}(\mathbf{x_0})\right]. \tag{9}$$

Here, the integration path goes from the reference point $\mathbf{x_0}$ to $\mathbf{x}$, as shown in Supplementary Fig. 1.

To verify the quasi-steady-state assumption, we compared the ratio of the inertial force to the elastic force per unit volume for solid-sphere impact:

$$R = \left| \frac{\rho_s \frac{\partial^2 u_z}{\partial t^2}}{\frac{\partial \sigma_{zz}}{\partial z}} \right|, \tag{10}$$

where ρ_s is the density of the PDMS gels. R is less than about 2% near the impact axis where the impact pressure is above the noise level. Consistent with the estimate, the impact pressure of solid spheres calculated based on the quasi-steady-state assumption quantitatively matches that of the finite-element simulations without the assumption (Fig. 2b of the main text). As the time scale of solid-sphere impact is much shorter than that of drop impact under comparable impact conditions, the quasi-steady-state assumption should work even better for drop impact. Lastly, it is worth of noting that the calculation of shear stress—the quantity most relevant to surface erosion—does not rely upon the quasi-steady-state assumption and therefore is immune to the errors associated with the assumption.

3 Theory and numerical solutions

The deformation of an elastic medium at small strains is described by the Navier-Lamé equation:

$$(\lambda + 2G)\nabla(\nabla \cdot \mathbf{u}) - G\nabla \times (\nabla \times \mathbf{u}) + \mathbf{F} = \rho_s \frac{\partial^2 \mathbf{u}}{\partial t^2}, \tag{11}$$

where $\lambda = E\nu/[(1+\nu)(1-2\nu)]$ is the Lamé coefficient and $G = E/[2(1+\nu)]$ is the shear modulus. ρ_s, E and ν are the density, Young's modulus and Poisson's ratio of the medium, respectively. $\mathbf{u} = (u_r, u_\theta, u_z)$ is the displacement of the medium in a cylindrical coordinate. $\mathbf{F}$ is the body force per unit volume. Since $\mathbf{F}$ is small compared with the impact stress, we ignore the term. In the cylindrical coordinate, an axisymmetric Navier-Lamé equation reduces to

$$(\lambda + 2G)\partial_r \left[\frac{1}{r}\partial_r(ru_r) + \partial_z u_z\right] + G\partial_z(\partial_r u_z - \partial_z u_r) = \rho_s \frac{\partial^2 u_r}{\partial t^2} \tag{12}$$

$$(\lambda + 2G)\partial_z \left[\frac{1}{r}\partial_r(ru_r) + \partial_z u_z\right] - G\frac{1}{r}\partial_r[r(\partial_r u_z - \partial_z u_r)] = \rho_s \frac{\partial^2 u_z}{\partial t^2}. \tag{13}$$

A full analytic solution that mechanically couples the dynamics of an impacting drop with a deformable elastic impacted substrate is prohibitively difficult from the mathematical point of view. To the best of our knowledge, no attempt has been made in solving such a complicated problem. To take the first step and tackle a relatively easier problem, we determine the deformation of the elastic medium under the impact of a liquid drop by adopting the theoretical prediction of the pressure and shear stress distributions of incompressible

drops on infinitely rigid substrates [3]. This approximation can be considered as the one-way coupling between the impacting drop and the impacted substrate, where the substrate deforms under the impact stress of the liquid drop but does not modify the impact stress. This is justified when the deformation of the substrate is small like in the case of our experiments, and its accuracy increases with the stiffness of the material. The semi-quantitative agreement between the measured shear stress distributions and the theoretical prediction (Eq. (3) of the main text) away from the singular region suggests that the one-way approximation can qualitatively capture the dynamics of impacted substrates under drop impact.

Under the one-way approximation, we apply the pressure and shear distributions of incompressible drops on infinitely rigid substrates without the effect of ambient air as the boundary conditions of Eqs. (12) and (13):

$$\sigma_{rz}|_{z=0} = \begin{cases} \sqrt{\frac{6}{\pi^3}\frac{1}{Re}}\rho U^2 \frac{2rD}{3UDt-2r^2} & \text{for} \quad r \leq r_t \\ 0 & \text{for} \quad r > r_t \end{cases} \tag{14}$$

$$\sigma_{zz}|_{z=0} = \begin{cases} \frac{3\sqrt{2}}{2\pi}\rho U^2 \frac{D}{\sqrt{3UDt-2r^2}} & \text{for} \quad r \leq r_t \\ 0 & \text{for} \quad r > r_t, \end{cases} \tag{15}$$

where ρ, U and D are the density, the impact velocity and the diameter of the liquid drop, respectively. $r_t = \sqrt{6UDt}/2$ is the position of the turning point, where the pressure and shear stress diverge and exhibit the finite-time singularity [3]. Note that the shear stress $\sigma_{rz}|_{z=0}$ is a factor $1/\sqrt{Re} \sim 7 \times 10^{-3}$ smaller than the impact pressure $\sigma_{zz}|_{z=0}$. Thus, the impact pressure is the leading factor controlling the deformation of the impacted substrate (Fig. 4d-f and the associated discussion in the main text).

To make the equations dimensionless, we propose the following scaling, $t \sim DM^2/U$, $u \sim DM^2$, $(r, z) \sim DM$, $(\lambda, G) \sim E$ and $\sigma \sim ME \sim E^{1/2}(\rho U^2)^{1/2}$. Here, we introduce a Mach number $M \equiv U\sqrt{\rho_s/E}$, which compares the speed of the impacting drop U with the characteristic speed of sound of the elastic medium $\sqrt{E/\rho_s}$. For example, the speed of the body P wave is $v_p = C\sqrt{E/\rho_s}$, where $C = \sqrt{(1-\nu)/[(1+\nu)(1-2\nu)]} = 4.1 \sim \mathcal{O}(1)$ for an elastic material with Poisson's ratio $\nu = 0.49$. $M = 0.292$ in our experiments.

The proposed scaling yields the following boundary value problem of partial

differential equations:

$$(\lambda + 2G)\partial_r \left[\frac{1}{r}\partial_r(ru_r) + \partial_z u_z\right] + G\partial_z(\partial_r u_z - \partial_z u_r) = \frac{\partial^2 u_r}{\partial t^2} \quad (16)$$

$$(\lambda + 2G)\partial_z \left[\frac{1}{r}\partial_r(ru_r) + \partial_z u_z\right] - G\frac{1}{r}\partial_r[r(\partial_r u_z - \partial_z u_r)] = \frac{\partial^2 u_z}{\partial t^2}, \quad (17)$$

and

$$\sigma_{rz}|_{z=0} = \begin{cases} \sqrt{\frac{6}{\pi^3}\frac{1}{Re}\frac{\rho}{\rho_s}\frac{2r}{3t-2r^2}} & \text{for} \quad r \leq r_t \\ 0 & \text{for} \quad r > r_t \end{cases} \quad (18)$$

$$\sigma_{zz}|_{z=0} = \begin{cases} \frac{3\sqrt{2}}{2\pi}\frac{\rho}{\rho_s}\frac{1}{\sqrt{3t-2r^2}} & \text{for} \quad r \leq r_t \\ 0 & \text{for} \quad r > r_t, \end{cases} \quad (19)$$

where all the quantities are now dimensionless with the turning point at $r_t = \sqrt{6t}/2$. The resulting equations and boundary conditions are independent of M in terms of the scaled variables. The scaling suggests that the shear force should scale as $F_d \sim \sigma D^2 \sim E^{1/2}(\rho U^2)^{1/2}D^2$, matching our experimental measurements at different E (Fig. 5b of the main text). Note that the density ratio between the liquid and the gel $\rho/\rho_s \sim \mathcal{O}(1)$ in our experiments.

We numerically solve Eqs. (16) and (17) with the boundary conditions Eqs. (18) and (19) using the finite element method (Methods). To avoid the singularity at r_t, we impose a small cut-off δ:

$$\sigma_{rz}|_{z=0} = \begin{cases} \sqrt{\frac{6}{\pi^3}\frac{1}{Re}\frac{\rho}{\rho_s}\frac{2r}{3(1+\delta)t-2r^2}} & \text{for} \quad r \leq r_t \\ 0 & \text{for} \quad r > r_t \end{cases} \quad (20)$$

and

$$\sigma_{zz}|_{z=0} = \begin{cases} \frac{3\sqrt{2}}{2\pi}\frac{\rho}{\rho_s}\frac{1}{\sqrt{3(1+\delta)t-2r^2}} & \text{for} \quad r \leq r_t \\ 0 & \text{for} \quad r > r_t. \end{cases} \quad (21)$$

We choose $\delta = 0.1$ for our numerical simulations, as a good convergence of solutions is achieved for the chosen spatial resolution.

The solutions of the radial and vertical displacements of the surface of the elastic medium, $u_r(r, z = 0, t)$ and $u_z(r, z = 0, t)$, are shown in Supplementary Figs. 2a and b. The in-phase propagation of the disturbance of u_r and u_z are the characteristic feature of the surface Rayleigh wave. The wave emerges around $t_c \approx 0.1$, consistent with the experimental observation. Moreover, the surface wave is sharp and concentrated with a well-defined peak propagating along the

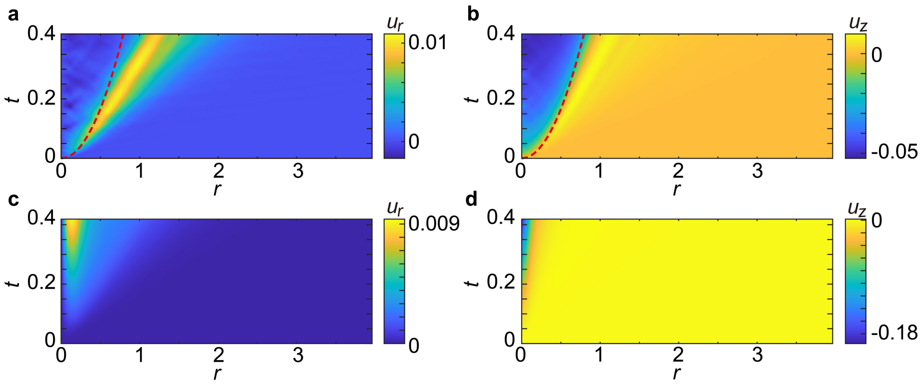

Supplementary Figure 2: Numerical solutions of the surface wave induced by the impact of a liquid drop and by the impact of a solid sphere. **a** and **b** The kymograph of the radial displacement of the impacted surface $u_r(r,t)$ and the vertical displacement of the impacted surface $u_z(r,t)$ induced by the impact of a liquid drop. **c** and **d** The kymograph of $u_r(r,t)$ and $u_z(r,t)$ induced by the impact of a solid sphere. $u_r(r,t)$ and $u_z(r,t)$ are normalized by the diameter of the impactors D. The red dashed lines in **a** and **b** indicate the position of the turning point where pressure and shear stress reach maximum. In contrast, the pressure maximum is stationary and fixed at $r = 0$ for the solid-sphere impact in **c** and **d**.

surface radially at a speed V_R, agreeing with the theoretical prediction of the speed of the Rayleigh wave. This sharp wave arises from the resonance when the speed of the stress maxima is close to the speed of the Rayleigh wave near t_c. The surface wave decays much slower than the body wave and therefore dominates the surface disturbance in the far field.

To compare with the surface wave induced by the impact of a solid sphere, we apply the Hertzian contact force in the dimensionless form [4]

$$F = \frac{2\sqrt{2}}{3}\frac{M^2}{1-\nu^2}t^{3/2} \tag{22}$$

on the surface of the elastic medium over a small region of $r_0 = 0.1$ around the impact axis. Here, we assume that the deformation of the solid sphere is negligible compared with that of the elastic medium and the vertical displacement $x \approx Ut$ at short times based on the experimental observation [5]. In comparison with the impact stress of liquid drops (Eqs. (18) and (19)), Eq. (22) is stationary in space and does not exhibit either the finite-time singularity or the shock front associated with the turning point at small t. With a stationary

stress maximum, the resonance in drop impact does not occur in solid-sphere impact either. Supplementary Figures 2c and d show the numerical solutions of the surface displacements, $u_r(r, z = 0, 0)$ and $u_z(r, z = 0, 0)$, of solid-sphere impact. Different from the shock-induced sharp surface wave of drop impact, the Rayleigh wave of solid-sphere impact emits continuously from $r = 0$ upon the impact at $t = 0$ and is much broader and more diffusive in nature.

Supplementary references

[1] Hall, M. S., Long, R., Hui, C.-Y. & Wu, M. Mapping three-dimensional stress and strain fields within a soft hydrogel using a fluorescence microscope, *Biophys. J.* **102**, 2241–2250 (2012).

[2] Belytschko, T., Lu, Y. Y., & Gu, L. Element-free galerkin methods, *Int. J. Numer. Methods Eng.* **37**, 229–256 (1994).

[3] Philippi, J., Lagrée, P.-Y., & Antkowiak, A. Drop impact on a solid surface: short-time self-similarity, *J. Fluid Mech.* **795**, 96–135 (2016).

[4] Landau, L. D. & Lifshitz, E. M. *Theory of Elasticity, 3rd ed.* (Butterworth-Heinemann, 1986).

[5] Gordillo, L., Sun, T.-P. & Cheng, X. Dynamics of drop impact on solid surfaces: evolution of impact force and self-similar spreading, *J. Fluid Mech.* **840**, 190–214 (2018).
